# Do trends in the prevalence of overweight by socio-economic position differ between India’s most and least economically developed states?

**DOI:** 10.1186/s12889-019-7155-9

**Published:** 2019-06-20

**Authors:** Shammi Luhar, Poppy Alice Carson Mallinson, Lynda Clarke, Sanjay Kinra

**Affiliations:** 1 Department of Population Health, London School of Hygiene and Tropical Medicine, Room G81, LSHTM, Keppel St, Bloomsbury, London, WC1E 7HT UK; 2 Department of Non-Communicable Disease Epidemiology, London School of Hygiene and Tropical Medicine, London, UK

**Keywords:** State economic development, India, Overweight, Socioeconomic status, Urban/rural

## Abstract

**Background:**

India’s economic development and urbanisation in recent decades has varied considerably between states. Attempts to assess how overweight (including obesity) varies by socioeconomic position at the national level may mask considerable sub-national heterogeneity. We examined the socioeconomic patterning of overweight among adults in India’s most and least economically developed states between 1998 and 2016.

**Methods:**

We used state representative data from the National Family Health Surveys from 1998 to 99, 2005–06 and 2015–16. We estimated the prevalence of overweight by socioeconomic position in men (15–54 years) and women (15–49 years) from India’s most and least economically developed states using multilevel logistic regressions.

**Results:**

We observed an increasing trend of overweight prevalence among low socioeconomic position women. Amongst high socioeconomic position women, overweight prevalence either increased to a smaller extent, remained the same or even declined between 1998 and 2016. This was particularly the case in urban areas of the most developed states, where in the main analysis, the prevalence of overweight increased from 19 to 33% among women from the lowest socioeconomic group between 1998 and 2016 compared to no change among women from the highest socioeconomic group. Between 2005 and 2016, the prevalence of overweight increased to similar extents among high and low socioeconomic status men, irrespective of residence.

**Conclusions:**

The converging prevalence of overweight by socioeconomic position in India’s most developed states, particularly amongst urban women, implies that this subpopulation may be the first to exhibit a negative association between socioeconomic position and overweight in India. Programs aiming to reduce the increasing overweight trends may wish to focus on poorer women in India’s most developed states, amongst whom the increasing trend in prevalence has been considerable.

**Electronic supplementary material:**

The online version of this article (10.1186/s12889-019-7155-9) contains supplementary material, which is available to authorized users.

## Background

The considerable rise in the prevalence of overweight (including obesity) in India, where over a billion people reside [1–4], presents a serious public health concern given the association of overweight with increased non-communicable disease (NCD) risk [5].

In the early stages of economic development and urbanisation, overweight and obesity prevalence tends to be higher among individuals of a higher socioeconomic position (SEP), arguably due to an increased financial capability to meet and exceed nutritional requirement [6–9]. As societies develop economically, the prevalence of overweight increases among the poor and rural population [6–14].

Since India’s economic liberalisation in the early 1990 [15], economic growth has not been uniformly distributed across the country. In addition to considerable heterogeneity in culture, customs and diet, the current levels of economic development between India’s states varies substantially. For example, the Gross Domestic Product of Delhi is eight times greater than that of the state of Bihar [16]. Consequently, the prevalence of overweight, and the extent of the increase in its prevalence in recent decades, varies considerably sub-nationally [1–3]. For instance, in Bihar, the prevalence of overweight among women increased from 3.7 to 11.7% (an absolute increase of 8%) between 1998 and 2016, whereas in Delhi, the prevalence increased from 12 to 33.5% over the same period (an absolute increase of 21.5%) [1]. However, little is known about variation in the sub-national socioeconomic patterning of overweight.

In this paper, we aimed to understand how recent trends in the association between overweight and SEP differ between India’s most and least economically developed states between 1998 and 2016, a period in which India’s Gross Domestic Product per capita quadrupled from US$432 to US$1750 [17]. The main rationale for this study was to unmask subnational heterogeneity in trends in the association of overweight and SEP in India not observed when analysing national trends. Demonstrating this would imply that national-level trends may not be generalisable at a subnational level [18]. A study of this nature is of importance as health policy is dictated at the state level; therefore, estimating the prevalence by state development and urban and rural areas may highlight different immediate health policy priorities between less and more developed states.

We conducted secondary analysis, using repeated cross-sections from state-representative data from 1998 to 2016 to estimate the prevalence of overweight in India by SEP in the five most and least economically developed states in India. In more economically developed societies, there is usually higher prevalence of overweight among poorer individuals where, for instance, there is a higher exposure to relatively cheaper fatty foods [6, 9, 19]. This is more likely to be the case in urban areas, where risk factors for overweight are usually much greater. We therefore hypothesise that in India’s most developed states, we will observe a considerable increase in the prevalence of overweight among lower SEP individuals and relatively smaller increases among higher SEP individuals. On the other hand, in India’s least developed states, we expected to find larger increases among higher SEP individuals, compared to lower SEP individuals. This is supported by the fact that poorer individuals in societies with lower levels of economic development are more likely to be unable to afford to meet nutritional requirements, whereas the relatively rich may be more exposed to overweight due to a greater access to excess food [6, 9].

### Data

We used the National Family Health Survey (NFHS) Surveys 2 (1998–99), 3 (2005–06) and 4 (2015–16). All three surveys collected health and demographic data on women aged 15–49, whereas surveys 3 and 4 collected data on men aged 15–54. The sampling method was designed to include a nationally-representative sample of individuals within a nationally-representative sample of households. Additionally, in India, the NFHS surveys are also representative at the level of the state.

The NFHS surveys select rural and urban samples separately. Specifically, in rural areas in all three waves analysed, rural samples were selected using two-stage sampling, whereby the first stage involved selecting primary sampling units (PSUs), or villages, with a probability proportional to size (PPS), and the second stage involved selecting random households from each village. In urban areas, NFHS 2 and 3 used a slightly different sampling procedure to the one in NFHS 4. In NFHS 2 and 3, three-stage sampling was adopted whereby in the first stage wards were selected with a PPS, in the second random census enumeration blocks (CEB) were chosen in each ward and, in the third, random households were chosen from each CEB [2, 3] On the other hand, NFHS 4 adopted a two-stage approach in urban areas, whereby CEBs served as the PSU, selected using a PPS, and households from each PSU randomly selected. Were a PSU to contain fewer than 40 households, the PSU was joined to the nearest PSU. The 2011 census helped determine the sampling frame in NFHS 4 [1].

In all three surveys Interviews used a uniform questionnaire and were conducted by survey teams. A woman’s eligibility for the survey was determined by whether they were between ages 15–49 and, for the NFHS 3 and 4, whether they spent the previous night in the selected households. Men aged 15–54 in the households were eligible for the Men’s survey in NFHS 3. Of the selected households in NFHS 4, a random sample of households were selected to determine eligibility for the men’s survey [1].

In India there are currently 36 States/Union Territories. We restricted our analysis to states that have been in existence since the collection of the NFHS 2 survey. States created between the surveys were not considered in the analysis. We selected five states to indicate the most and least developed states as the study aimed to demonstrate a divergence in the trends in their socioeconomic patterning. Our primary objective was to highlight variation in trends in the socioeconomic patterning of overweight within India. We therefore chose not to include all the states in India as this would lead to the inclusion of states that are closer to the average level of per capita net state domestic product for India. As a result, we would risk placing states at similar levels of economic development in the Most and Least developed states categories, consequently underestimating the extent of the variation in trends.

Our classification of states was based on the per capita net state domestic product (PCNSDP) in 2014–15 using the base year 2011–12. The most economically developed states were Goa, Maharashtra, Sikkim, Haryana and Kerala with a PCNSDP ranging from ₹112,444 to ₹241,081, compared to an all India average of ₹72,805. The least economically developed states included Bihar, Assam, Uttar Pradesh, Manipur, and Madhya Pradesh with NSDPPC ranging from ₹23,223 to ₹44,809 [20]. We limited our sample to non-pregnant women, whose inclusion could bias the associations we sought to identify. This left a total of 96,365 women and 18,729 men in the most developed states category, and 289,200 women and 54,669 men, respectively, in the least developed states category.

As NFHS-2 only sampled ever-married women, we restricted our samples in 2005–06 and 2015–16 to this population to allow the comparability of the study population across surveys. Additionally, respondents with missing height and weight data were also omitted from the sample, leaving 76,050 women (12,168 in 1998–99; 14,000 in 2005–06; 49,882 in 2015–16) and 18,729 men (8518 in 2005–06 and 10,211 in 2015–16) as the study population in the most economically developed states, and 213,195 women (22,266 in 1998–99; 20,459 in 2005–06; and 170,470 in 2015–16) and 54,669 men (19,377 in 1998–99; and 35,292 in 2015–16) in the least economically developed states. As multi-stage sampling approaches were adopted in the collection of the NFHS, we included the sampling weights included in the data set to account for unequal selection probabilities.

### Outcome

We used the Body Mass Index (BMI) variable included in the surveys (measured as the respondent’s weight divided by the square of their height) to separate individuals into two groups: overweight (BMI over 24.99 kg/m^2^), and not overweight (BMI 24.99 kg/m^2^ or under). This categorisation is based on the WHO’s recommended cut-offs for BMI classification [5]. Rather than split the continuous BMI measure into multiple subcategories of overweight, we used this classification as the main aim of the paper was to analyse trends in excess adiposity, and research has found an elevated risk of NCDs and mortality beyond a BMI of 24.99 kg/m^2^ [21, 22]. We did not use a continuous measure of nutritional status, as observed population-level increases in BMI we would expect to observe over the study period could be driven by a both individuals moving into overweight categories, and individuals moving from underweight to normal weight; the latter of which does not capture increases in excess adiposity.

Height and weight information on women aged 15–49 in NFHS-2, 3 and 4, and men aged 15–54 in NFHS-3 and 4, were collected by specially trained investigators. A solar-powered SECA digital scale was used to measure the weight of respondents, with the NFHS-2 report claiming an accuracy of ±100 g. The height of respondents in NFHS-2 and 3 was measured using a measuring board designed for use in survey data collection. In NFHS-4, the Seca 213 stadiometer was used to collect respondent’s height information [1–3].

### Independent variables

#### Exposure of interest

We used a measure of educational attainment as our primary indicator of SEP. This was based on the answer to a question regarding the number of completed years of schooling, and respondents were assigned to one of the following education categories: No Education (0 years); Primary Education (1–5 years); Secondary Education (6–12 years); and Higher Education (12+ years). Higher levels of education can increase earning capability, along with the accumulation of employable skills, both of which make it a suitable proxy for SEP.

For sensitivity analysis we verified our results using a standard of living (SoL) asset-based index as an alternative measure of SEP. In surveys, measures of SEP are seldom examined in isolation, as one measure cannot adequately describe all socioeconomic differences in a health outcome [23]. As education and SoL capture different aspects of SEP, the pathways through which it is associated with overweight may also differ. For example, those with high education may work in more sedentary jobs [6–9], increasing their risk of overweight, whereas SoL may be positively associated with overweight through determining the ability to afford excess food [6–9]. Some suggest that in low/middle income settings, where there is a substantial informal employment sector and earnings not in the form of monetary enumeration, household income may not be an appropriate measure of SEP. Rather, the stock of assets may be more reliable [24]. Data on household income to proxy SEP is likely to be very sensitive to seasonal fluctuations in repeated cross-sections and may not capture the true level of wealth of the household. Additionally, in transitioning societies, it may be more common to receive income ‘in-kind’ rather than monetary enumeration [25], and households may draw money from multiple sources [24], limiting the ability for respondents to adequately recall all income in a questionnaire.

We created our own SoL index using principal components analysis (PCA) after pooling the household surveys over time. The inputs we used into the PCA included information on the household’s stock of assets, their access to services, and other household characteristics. We completed this process for urban and rural areas separately due to differences in the importance of different assets between urban and rural residents. The percentage of respondent households in urban and rural areas by characteristics used to build the SoL index in each survey is presented in Additional file 1: Table S1. We then ranked households based on this new index and assigned the first, second and last third of the weighted sample a SoL classification of ‘Higher’, ‘Medium’ or ‘Lower’ Standard of Living (SoL).

We examined the validity of the SoL index we created by comparing the ranking of households using the index from the pooled data, within one survey, and the survey-specific wealth index already included in the data. The correlation coefficient in each of the three surveys used was greater than 0.95, suggesting a very strong agreement with our measure and the household rankings determined the survey-specific index.

### Covariates

Our final models were adjusted for the respondent’s age (15–29; 30–39; and 40–49 (40–54 for men)) and marital status. Marital status was categorised as either ‘currently married’ or ‘not currently married’ and was included as married individuals have been found to be at higher risk of being overweight [26]. We would have also preferred to control for the respondent’s occupation. Higher prevalence of overweight may be expected to be observed among individuals in more sedentary jobs [6–9], and sedentary labour may be expected to be more prevalent among higher SEP individuals. However, it was not possible to control for occupation in our research due to the fact that it was collected on a very limited subsample of the respondents in NFHS 4 (approximately 5% of women in the NFHS-4 national sample).

## Methods

In our preliminary analysis, we calculated the weighted prevalence of overweight in each strata of the education SEP variable, separately for India’s most and least developed states, by sex and urban/rural residence. We then calculated the ratio of the prevalence in the highest educational category to the lowest in each survey.

In order to account for the hierarchical nature of the data, in our main analysis we fitted multilevel logistic regressions with PSU-level random intercepts, for each sex, and urban/rural residence, separately. Failure to account for this deliberate clustering at the sampling stage of the data collection process would have caused us to underestimate the standard errors of our results. We used survey-specific interaction terms to estimate the log odds ratio of overweight in each category of our SEP exposure variables, relative to the lowest category of each SEP variable, in each survey. We monitored changes in standard errors of the main SEP exposure variable in order to determine whether there was multicollinearity of the main exposure with added covariates. Coefficients from the adjusted models were subsequently converted to a predicted prevalence, with 95% confidence intervals, to make the results easier to interpret.

## Results

The characteristics of respondents in the surveys used are presented in Table 1. In both the most and least developed states, the percentage of women with secondary education and in the Higher SoL category is higher in later surveys, compared to earlier ones. On the other hand, the percentage of women with no education and in the Lower SoL category decreases over the surveys. For example, in the most developed states, the percentage of women in the Higher SoL category increases from 16 to 65% between NFHS 2 and 4, whereas the percentage in the Lower SoL category decreases from 44 to 9%. The percentage of respondents classified as overweight increases in each successive survey. The largest increase was observed among women in the least developed states, where the percentage of overweight respondents increased from 6 to 19% between NFHS 2 and 4. Similar trends are found even when we do not limit our sample to non-pregnant and ever-married women (Additional file 1: Table S2).

**Table 1:** Percentage* and number of study participants by key variables in each of the surveys

	*Women*		*Women*		*Women*		*Men*		*Men*	
	*1998–99*		*2005–06*		*2015–16*		*2005–06*		*2015–16*	
	*%*	*Freq*	*%*	*Freq*	*%*	*Freq*	*%*	*Freq*	*%*	*Freq*
*Most developed states*										
*Overweight*	18.24	2220	24.12	3377	27.26	13,598	14.19	1658	24.15	3323
*Age 15–29*	38.44	4677	33.84	4737	33.11	16,518	48.73	6041	44.76	7056
*Age 30–39*	36.05	4386	38.90	5446	36.15	18,033	25.58	3145	25.76	4054
*Age 40–49 (54 males)*	25.52	3105	27.26	3817	30.73	15,331	25.69	3088	29.48	4454
*Urban*	40.35	4910	49.40	6916	35.40	17,656	59.26	7294	37.31	5788
*Rural*	59.65	7258	50.60	7084	64.60	32,226	40.74	4980	62.69	9776
*No Education*	32.10	3905	23.64	3309	19.30	9626	6.98	1010	6.15	1292
*Primary*	18.85	2294	15.43	2160	13.57	6771	13.85	1741	10.20	1856
*Secondary*	35.93	4371	50.21	7030	54.48	27,178	63.25	7624	64.47	9784
*Higher*	13.13	1597	10.72	1501	12.64	6307	15.92	1895	19.18	2632
*Lower SoL*	44.28	5373	30.39	4250	8.97	4447	36.43	4524	8.74	1693
*Middle SoL*	39.74	4822	34.77	4862	26.18	12,985	34.52	4335	24.97	4418
*Higher SoL*	15.99	1940	34.84	4873	64.85	32,161	29.05	3406	66.29	9351
*Married*	93.41	11,366	93.24	13,053	94.16	46,971	58.96	7339	62.11	9961
*Not Married*	6.59	802	6.76	947	5.84	2911	41.04	4935	37.89	5603
*Least developed states*										
*Overweight*	6.00	1336	14.67	3002	18.51	31,546	9.56	1853	12.88	4544
*Age 15–29*	46.49	10,352	39.32	8044	36.88	62,868	51.00	9882	49.02	17,300
*Age 30–39*	32.19	7168	35.94	7353	34.71	59,173	24.87	4819	24.16	8527
*Age 40–49 (54 males)*	21.32	4746	24.74	5062	28.41	48,429	24.13	4676	26.82	9465
*Urban*	20.50	4564	40.75	8338	22.98	39,174	46.62	9034	27.55	9724
*Rural*	79.50	17,702	59.25	12,121	77.02	131,296	53.38	10,343	72.45	25,568
*No Education*	62.67	13,951	48.88	9999	44.43	75,735	16.12	3122	16.49	5820
*Primary*	14.22	3166	13.52	2766	14.29	24,364	13.77	2667	14.14	4990
*Secondary*	16.14	3594	28.94	5921	33.91	57,798	53.78	10,416	55.35	19,535
*Higher*	6.96	1550	8.66	1771	7.38	12,573	16.34	3164	14.02	4947
*Lower SoL*	69.21	15,333	55.29	11,301	30.03	47,214	51.81	10,032	28.21	9164
*Middle SoL*	24.98	5534	27.13	5546	38.52	60,571	28.53	5525	38.99	12,667
*Higher SoL*	5.81	1288	17.58	3594	31.45	49,457	19.66	3806	32.80	10,654
*Married*	94.24	20,984	94.61	19,356	95.42	162,655	60.84	11,788	62.35	22,006
*Not Married*	5.76	1282	5.39	1103	4.58	7815	39.16	7589	37.65	13,286

^*^All percentages are based on unweighted proportions

**Fig. 1:**
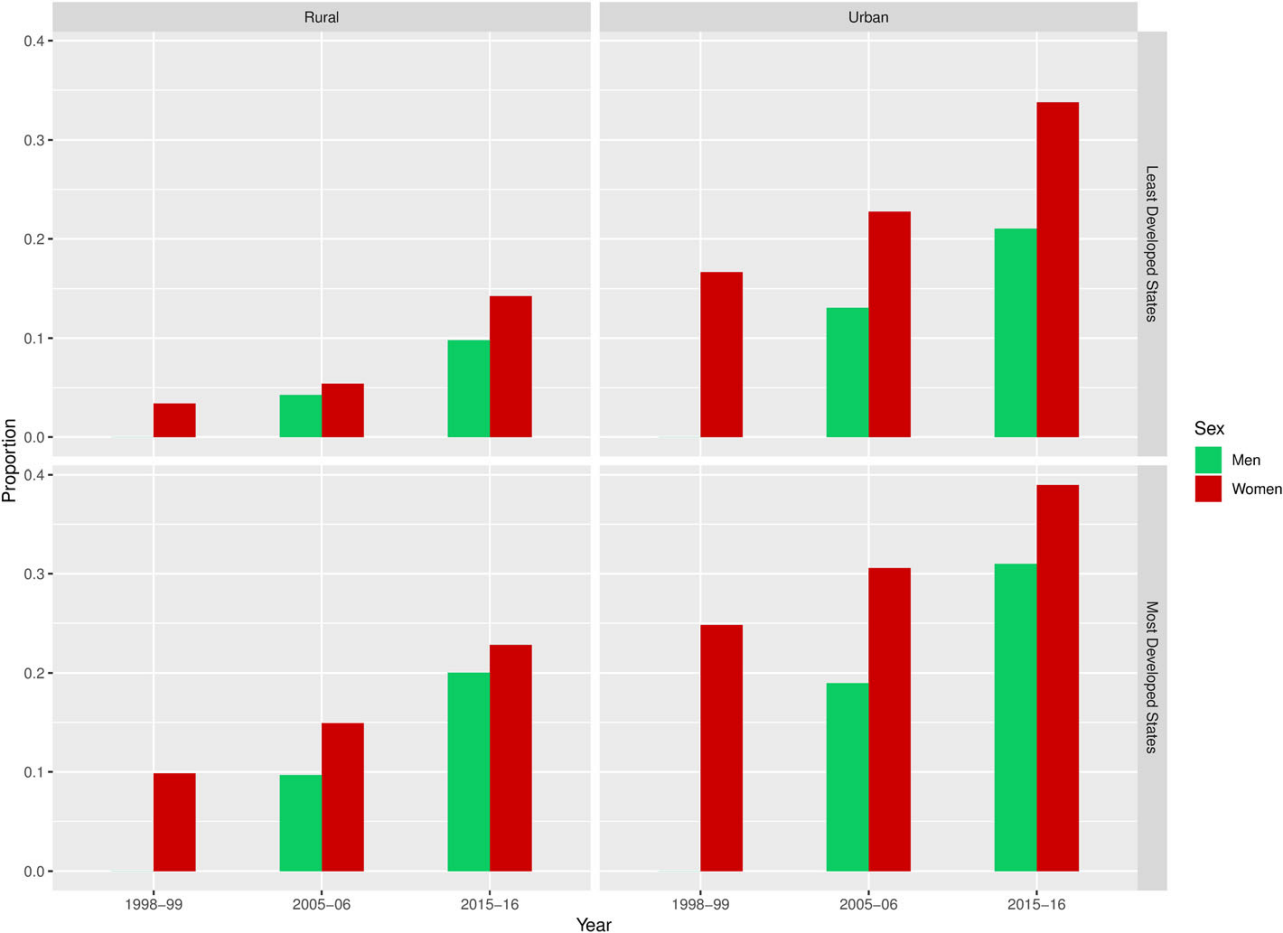
Prevalence of overweight in 1998–99, 2005–06 and 2015–16 in urban and rural India

In our preliminary analysis we found a consistent trend of increasing prevalence of overweight in both India’s most and least developed states. This trend was found amongst both men and women in urban and rural areas (Fig. 1). As expected, the most developed states generally had a higher overall level of overweight prevalence compared to the least developed states, and especially in urban areas and among women.

We found a higher relative increase in overweight prevalence in India’s least developed states. Whereas the prevalence among urban women doubled from 17 to 34% in the least developed states between 1998 and 2016, the prevalence increased from 24 to 39% among urban women in the most developed states. Similarly, in among rural women, overweight increased nearly five-fold, from 3 to 14%, in the least developed states, compared to an increase from 10 to 23% in the most developed states.

Although the prevalence of overweight increased among individuals of all educational attainments, the extent of the increase in prevalence over the study period was consistently highest among those with lower levels of education (Table 2). This was reflected in a declining the ratio of prevalence among those with higher education compared to those with no education. For example, in urban areas of India’s most developed states, the prevalence of overweight was 5.14 times higher among highly educated women than women with no education in 1998–99 compared to 1.79 times higher in 2015–16.

**Table 2:** Percentage* of respondents classified as overweight by education level

		*Most developed states*					*Least developed states*				
		*Women*			*Men*		*Women*			*Men*	
		*1998–99*	*2005–06*	*2015–16*	*2005–06*	*2015–16*	*1998–99*	*2005–06*	*2015–16*	*2005–06*	*2015–16*
Education**											
*Rural*	*No Education*	4.09	7.90	16.52	4.31	13.85	2.43	3.69	11.74	2.21	6.29
	*Primary Education*	10.07	15.46	20.73	8.13	19.81	4.44	6.23	14.31	1.64	7.42
	*Secondary Education*	13.74	18.43	24.51	9.41	19.31	6.33	9.86	17.51	4.45	9.88
	*Higher Education*	20.99	28.20	29.51	19.27	25.54	11.01	16.47	22.38	15.37	18.57
	*Ratio (Higher: No education)*	5.14	3.57	1.79	4.47	1.84	4.53	4.46	1.91	6.94	2.95
Education**											
*Urban*	*No Education*	16.62	21.27	34.77	11.71	15.90	8.98	14.38	28.74	4.61	14.59
	*Primary Education*	21.10	25.62	37.07	13.61	29.46	13.05	19.92	29.68	7.00	15.28
	*Secondary Education*	25.42	32.54	39.79	17.70	28.43	18.87	27.74	34.80	11.24	19.42
	*Higher Education*	35.16	37.70	40.21	27.52	40.79	29.34	37.75	42.10	26.39	30.55
	*Ratio (Higher: No education)*	2.12	1.77	1.16	2.35	2.56	3.27	2.63	1.47	5.72	2.09

^*^All percentages were calculated using sampling weights

**Chi-squared test *p* value of the variable’s association with overweight: *p* < 0.001

Notably, the smallest ratio was reported among women in 2015–16 in urban areas of the most developed states, whereas the highest ratio among women was found in rural areas of the least developed states. Among men, the lowest ratio was found among rural residents in the most developed states, whereas the highest was found in rural areas of India’s least developed states.

**Fig. 2:**
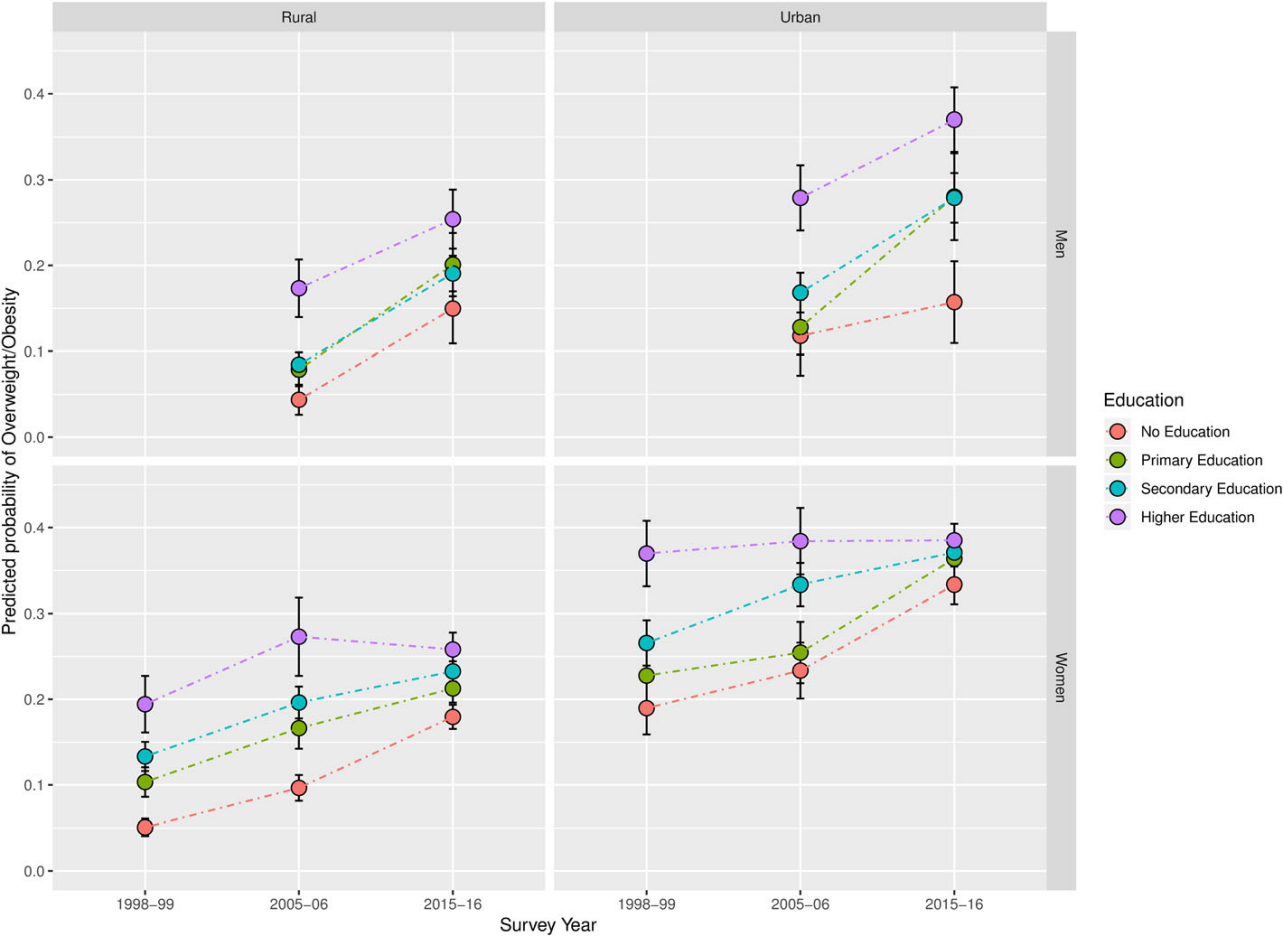
Predicted prevalence of overweight by Education Level between 1998 and 99 and 2015–16 in India’s most developed states

**Fig. 3:**
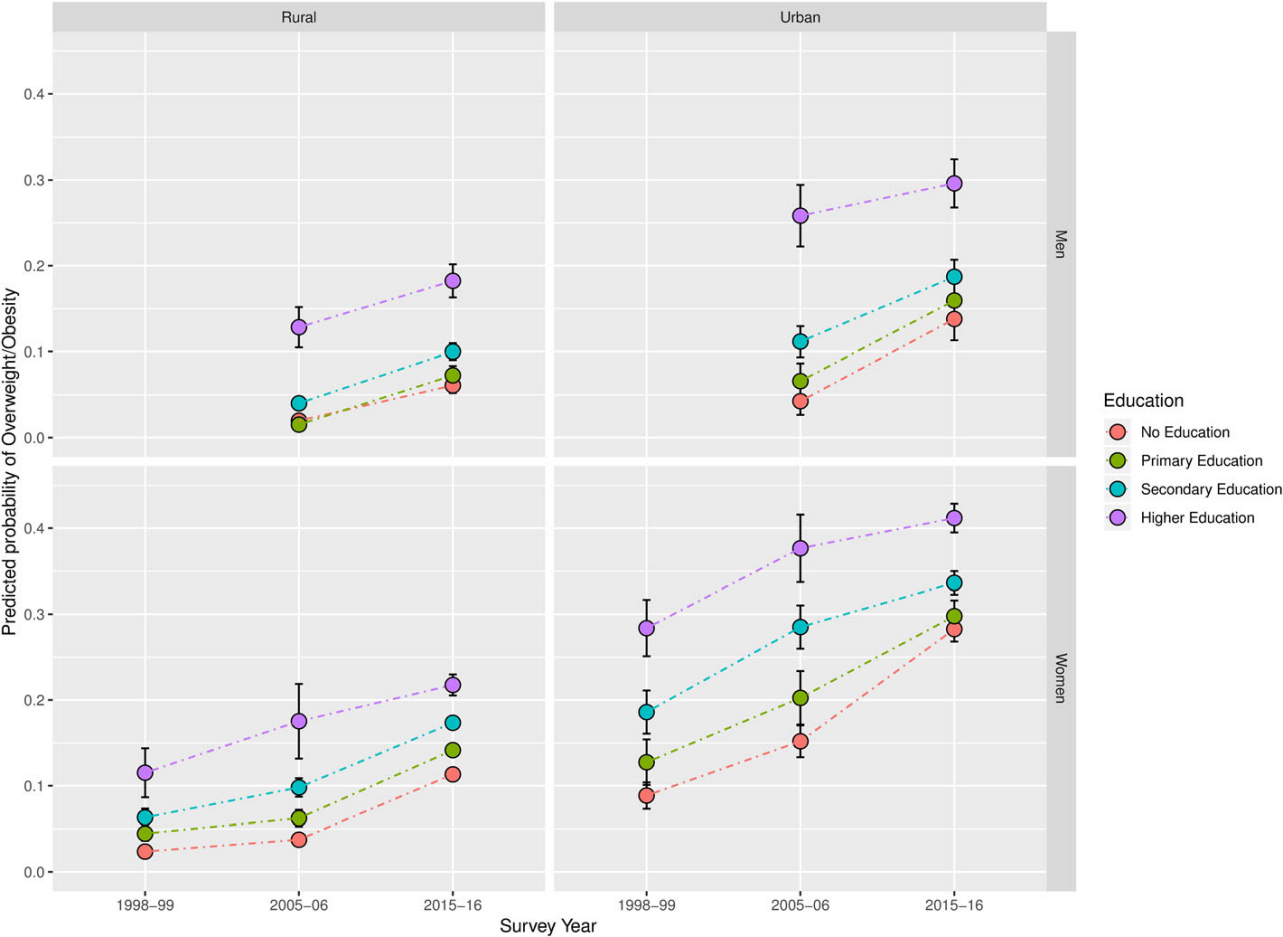
Predicted prevalence of overweight by Education Level between 1998 and 99 and 2015–16 in India’s least developed states

In our adjusted analysis we found that the difference in prevalence between the highest and lowest SEP category generally declined among women between 1998 and 99 and 2005–06 (Figs. 2 and 3). The largest decline in this difference was among women in the most developed states, where we observed substantial increases among women with low educational attainment between 1998 and 2016, and no notable increase among women with higher education. In the least developed states, we observed increases in overweight prevalence among women of all educational attainments, however, the increases were to a greater extent among women with little or no education.

Although the overall prevalence of overweight is consistently higher among urban women than rural women, we found no notable differences between them in their socioeconomic patterning trends. We identified very limited evidence of a smaller difference of overweight prevalence between men with higher education and no education in 2015–16 compared to 2005–06.

### Sensitivity analysis

**Fig. 4:**
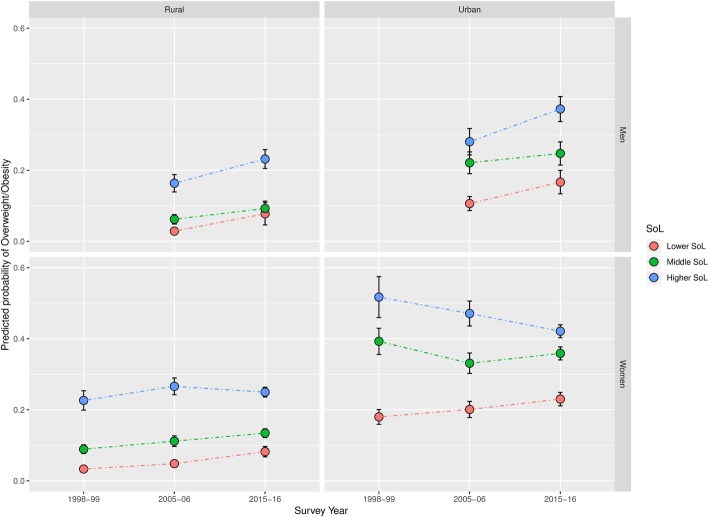
Predicted prevalence of overweight by SoL between 1998 and 99 and 2015–16 in India’s most developed states

**Fig. 5:**
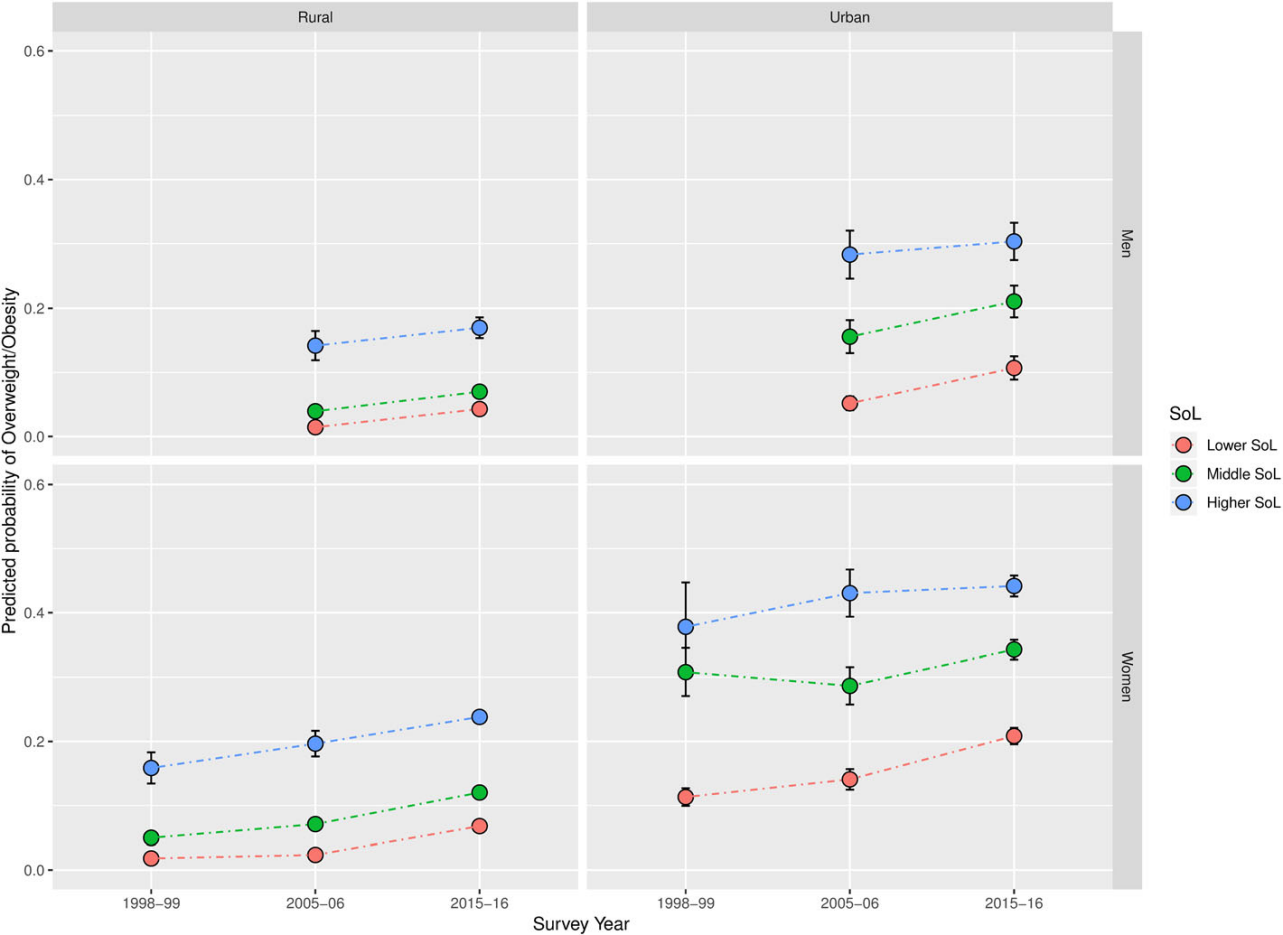
Predicted prevalence of overweight by SoL between 1998 and 99 and 2015–16 in India’s least developed states

Results of our sensitivity analysis are presented in Table 3, and in Figs. 4 and 5. Using the SoL index as the main exposure, shows a similar trend of a notable convergence of overweight prevalence across SEP among women, particularly in urban areas of India’s most developed states. Additionally, it supports the considerably more mixed trend of overweight patterning among men we identified when using education as the exposure of interest.

**Table 3:** Percentage* of respondents classified as overweight by Standard of Living

		*Most developed states*					*Least developed states*				
		*Women*			*Men*		*Women*			*Men*	
		*1998–99*	*2005–06*	*2015–16*	*2005–06*	*2015–16*	*1998–99*	*2005–06*	*2015–16*	*2005–06*	*2015–16*
SoL**											
*Rural*	*Lower SoL*	3.13	4.29	7.63	2.76	7.91	1.88	2.37	7.13	1.70	4.70
	*Middle SoL*	9.66	10.96	12.96	6.41	9.57	5.02	6.94	12.28	4.83	7.77
	*Higher SoL*	24.79	27.42	27.25	18.10	23.98	16.04	19.96	25.21	16.49	17.67
	*Ratio (Higher: Lower SoL)*	7.92	6.39	3.57	6.55	3.03	8.54	8.41	3.53	9.68	3.76
SoL**											
*Urban*	*Lower SoL*	16.62	19.6	26.48	10.98	19.88	11.48	13.58	20.36	5.55	11.15
	*Middle SoL*	39.06	32.62	38.53	23.36	27.48	33.06	29.27	34.72	17.19	21.69
	*Higher SoL*	49.64	46.5	44.45	29.04	38.76	38.29	43.79	44.83	31.13	30.69
	*Ratio (Higher: Lower SoL)*	2.99	2.37	1.68	2.64	1.95	3.34	3.22	2.20	5.61	2.75

^*^All percentages were calculated using sampling weights

**Chi-squared test *p* value of the variable’s association with overweight: *p* < 0.001

## Discussion

### Statement of findings

This study has found that the trends in the socioeconomic patterning of overweight in India varied between India’s most and least developed states between 1998 and 2016. When examining the difference between overweight prevalence in the highest and lowest SEP groups, we found a converging trend of overweight prevalence across SEP among women between 1998 and 2016. As expected, this trend amongst women was more pronounced in India’s most developed states, particularly in urban areas, however, similar trends were observed in the least developed states and in rural areas. The converging trend amongst women appears to be driven by relatively smaller increases, and in some cases a decline, in the prevalence of overweight in higher SEP groups compared to lower SEP groups. This convergence appears to be limited to women, as amongst men, we did not identify any notable convergence in the socioeconomic patterning of overweight between 2005 and 2016. Using SoL as the main exposure of interest, we found similar, albeit a more attenuated convergence in the socioeconomic patterning of overweight.

Few studies have examined sub-national variation in the association of SEP and overweight in countries that have undergone rapid economic development. The only similar study we identified in India used a standard of living index as the primary exposure and reported a similar converging trend in the prevalence of overweight across SEP in states with a high overall prevalence of. On the other hand, they identified a diverging trend in Standard of Living in states with a high prevalence of underweight amongst women between 1998 and 2006 [27]. Our more up-to-date examination of sub-national trends in additional subpopulations suggests a more nuanced picture of the socioeconomic patterning. Notably we find no evidence of a diverging trend of overweight across SEP in any of the subpopulations, and that there are notable differences in the trends between the sexes and urban and rural areas.

Although there still remains a positive association between SEP and overweight across all the subpopulations we analysed in India, studies in other countries have identified a negative association between SEP and excess weight in more economically areas of countries that have undergone rapid economic development. One study in Brazil found a positive association between obesity and per capita household income in both more and less economically developed regions of Brazil in 1974/75. By 1996/97 the association was negative in the more economically developed regions, whereas the positive association in the less developed regions persisted [28]. This suggests that Brazil’s more developed regions in 1996/97 may have been at a more advanced stage of the epidemiological transition than India’s most developed states currently. Other studies using measures of household income and educational attainment as the primary exposures and focusing on women in China’s most economically prosperous regions have also found a negative association between SEP and prevalence of overweight [29, 30].

We also identified a particularly notable convergence in the prevalence of overweight by SEP among women when compared to men. Other studies have identified similar differences by sex. One study in China found high-income men and women with low education to be at highest risk of obesity in an economically prosperous province [29]. Another study in China found that higher education was associated with lower odds of overweight among women and higher odds of overweight among men [30]. In South Korea, a country that experienced a remarkable pace of economic growth in previous decades [31], one study still found a positive association of income with obesity among men, whereas they found a negative association among women [32].

The increased capacity of higher SEP individuals to afford to consume excess food [6–9] is a commonly suggested reason as to why the association between overweight and SEP is positive in low- or low-middle-income countries like India. However, the smaller difference in overweight prevalence between lower and higher SEP women in India’s most developed states, particularly in the most recent period, may be due to an increased ability to afford expensive healthy foods and an increased level of health consciousness among higher SEP individuals [6, 33, 34]. On the other hand, particularly in India’s most developed states, lower SEP individuals may be increasingly able to afford cheap high calorie fatty foods [6, 35]*.*

Our study has some limitations. Firstly, we would have ideally liked to have used additional indicators of excess adiposity to complement our findings. BMI may be limited in that it cannot distinguish between lean mass and body fat, nor does it have information on the distribution of body fat, potentially making it an inaccurate measure of central adiposity [36]. However, other studies have shown a strong correlation of BMI with measures of central adiposity among Indians, such as waist circumference [37]. Consequently, we would not expect the trends we report to vary considerably between adiposity measures. Another possible limitation associated with our use of the BMI variable to inform our main outcome of interest is the potential difference in the body fat percentage at any given BMI between higher and lower SEP groups. Research amongst children from higher income countries have shown that lower SEP groups may have a higher percentage of body fat at any given BMI compared to higher SEP groups [14, 38]. Although this may be limited to high income societies, we are unable to verify the association of body fat and BMI in our data as the NFHS does not collect body fat information. Were a similar phenomenon observed in India, this would imply a more rapid convergence in the socioeconomic patterning of overweight in India than we have reported.

We limited our study population of women in 2005–06 and 2015–16 to ever-married women, as this was the sampled population in 1998–99. However, a slightly higher proportion of the sample was never-married in 2015–16 (22.5%) than in 2005–06 (19.5%). Additionally, the prevalence of overweight is lower among never-married than in currently married women (prevalence of overweight was 6.6% and 25% among never married and currently married women, respectively, in 2015–16). This may have led us to potentially overestimate the prevalence we reported for 2015–16 and therefore underestimate the extent of convergence overweight prevalence across SEP (see Additional file 1: Table S3).

There are some slight differences in the rankings of states by PCNSDP in 2005–06 and 1998–99 compared to in 2014–15. For example, in 2005–06, the state of Odisha had a slightly lower PCNSDP than Manipur. Additionally, Gujarat’s economy is 19% larger than Sikkim’s in 2005–06, however, Sikkim’s economy almost tripled within a decade [20]. These discrepancies are however, unlikely to change the overall message of the study, and instead inclusion of these states is expected make results more relatively conservative.

Another limitation of our study involves our use of the standard of living index based in part on the ownership of assets. Common criticisms of an asset-based index like the one we used, includes the fact that it makes little accommodation for the quality of assets [24, 39], potentially leading to misclassification of households. For instance, televisions in poorer households may only receive terrestrial transmission, whereas in higher SEP households may receive digital transmission. Despite this, the simple collection of asset ownership information is not expected to affect the variable substantially [39]. Additionally, as we used three broad SoL categories across a large data set, any misclassification is not expected to be substantial. Another criticism of asset indices is that certain assets are likely to have different importance between broad geographical areas. Although we attempted to remedy this to an extent by calculating separate SoL indices in urban and rural areas, the importance of some assets may still differ between other geographical levels of aggregation [18]. Despite these issues, asset-based indices offer an affordable and stable long-term measure of household wealth for large surveys in low-income settings [24]. Furthermore, our use of two different measures of SEP in this study ensures that we have captured a large portion of the avenues through which SEP and overweight are associated.

Finally, the cross-sectional nature of the data we used did not enable us to draw conclusions about the causal relationship between overweight and SEP. Although this was not an explicit study aim, such information may have enriched our understanding of the reasons as to why overweight is more prevalent among particular socioeconomic groups in India.

Despite these limitations, we use the most recent state representative data, making our findings both generalisable and the most current estimates of these trends.

Some have suggested that overweight is a ‘disease of affluence’ in low and low-middle income countries [40, 41]. We find evidence of a much more nuanced picture of the socioeconomic patterning of overweight, when we examine sub-national trends. Whereas it may be an appropriate description of the positive associations between SEP and overweight we identified, were the identified trends to continue especially among women in India’s more economically developed states, there may be a negative association in the coming years. We find no evidence that were past trends to continue, there would be any change to the socioeconomic pattering among men.

The markedly higher increase in overweight among lower SEP Indians will be an important consideration in the near future as state governments are already tasked with tackling the burden of infectious diseases within this demographic. A state-specific approach will be needed to face the challenge of raising general access to staple foods whilst simultaneously trying to lower demand for unhealthy foods [27, 42–44]. Additionally, attempts to close the difference in the association of overweight with SEP between men and women may wish to focus on improving health-related behaviors among men.

## Conclusion

Although the association between SEP and overweight is still positive, a continuation of past trends suggests that urban areas of the most developed states in India may be the first to show a negative association commonly seen in high-income countries. The success of policies to slow the increasing prevalence of overweight may depend on understanding how trends in socioeconomic patterning of overweight have developed and may continue to develop in the future.

## Additional file

Additional file 1: **Table S1**. Percentage of households with the following assets/characteristics by survey and urban/rural residence – presents the ownership of assets and household characteristics by urban and rural residence across India in the three NFHS surveys used. **Table S2**. Percentage of the full women’s sample (including pregnant and never-married women) in each strata of the SEP exposures and the outcome – presents the distribution of the data across the SEP variables and main outcome in the full sample of women. **Table S3**. Predicted prevalence of overweight from the regression model in India’s least developed states (using the full sample of women including pregnant and never married women) – demonstrates potential underestimation of the convergence in socioeconomic patterning of overweight in least developed states when including never-married women and pregnant women. (DOCX 17 kb)

## Data Availability

The datasets supporting the conclusions of this article are available in the Measure DHS repository, https://dhsprogram.com/data/available-datasets.cfm. Data is available from the MEASURE DHS project upon reasonable online request after submission of concept paper.

## Abbreviations

BMI: Body mass index
NCD: Non-communicable disease
PCNSDP: Per capita net state domestic product
SEP: Socioeconomic position
SoL: Standard of living
