# Supplementary material for: Do trends in the prevalence of overweight by socio-economic position differ between India’s most and least economically developed states?
Source: BMC Public Health. 2019 Jun 20;19:783. doi: 10.1186/s12889-019-7155-9 (PMC6585059; doi:10.1186/s12889-019-7155-9)
Supplement: Supplementary file 1 — Table S1. Percentage of households with the following assets/characteristics by survey and urban/rural residence – presents the ownership of assets and household characteristics by urban and rural residence across India in the three NFHS surveys used. Table S2. Percentage of the full women’s sample (including pregnant and never-married women) in each strata of the SEP exposures and the outcome – presents the distribution of the data across the SEP variables and main outcome in the full sample of women. Table S3. Predicted prevalence of overweight from the regression model in India’s least developed states (using the full sample of women including pregnant and never married women) – demonstrates potential underestimation of the convergence in socioeconomic patterning of overweight in least developed states when including never-married women and pregnant women. (DOCX 17 kb) [file 12889_2019_7155_MOESM1_ESM.docx]

Table A1 Percentage of households with the following assets/characteristics by survey and urban/rural residence

|  | ***1998-99*** | | ***2005-06*** | | ***2015-16*** | |
| --- | --- | --- | --- | --- | --- | --- |
| ***Asset*** | *Urban* | *Rural* | *Urban* | *Rural* | *Urban* | *Rural* |
| *Mattress* | 71.7 | 38.1 | 75.4 | 48.7 | 82.3 | 58.4 |
| *Pressure cooker* | 65.2 | 16.0 | 69.9 | 22.1 | 83.6 | 42.2 |
| *Chair* | 71.3 | 356.0 | 76.1 | 43.8 | 86.5 | 70.7 |
| *Cot/bed* | 86.1 | 79.4 | 86.3 | 81.2 | 88.5 | 88.3 |
| *Table* | 64.9 | 30.0 | 65.0 | 32.9 | 72.1 | 46.5 |
| *Clock/watch* | 90.1 | 57.5 | 91.0 | 71.4 | 90.8 | 71.4 |
| *Electric fan* | 82.2 | 31.4 | 84.7 | 38.6 | 95.1 | 69.1 |
| *Bike* | 53.5 | 45.7 | 50.1 | 51.6 | 45.0 | 55.9 |
| *Radio* | 53.2 | 32.2 | 38.9 | 27.0 | 10.3 | 7.0 |
| *Sewing Machine* | 35.5 | 11.9 | 30.9 | 12.6 | 33.5 | 19.0 |
| *Telephone* | 20.1 | 2.6 | 36.3 | 7.4 | 96.1 | 87.3 |
| *Refrigerator* | 28.8 | 3.7 | 33.5 | 6.6 | 54.2 | 16.4 |
| *Television (B+W)* | 44.8 | 17.0 | 25.6 | 18.7 | 3.1 | 3.5 |
| *Television (Colour)* | 27.3 | 3.5 | 51.5 | 12.5 | 86.0 | 51.5 |
| *Moped/Scooter/Motorcycle* | 25.0 | 6.0 | 30.5 | 10.8 | 51.5 | 30.3 |
| *Car* | 4.4 | 0.6 | 6.1 | 1.0 | 11.4 | 3.2 |
| *Water Pump* | 9.3 | 8.2 | 11.0 | 9.9 | 21.5 | 14.9 |
| *Thresher* | 0.7 | 2.5 | 0.4 | 2.2 | 0.6 | 1.9 |
| *Tractor* | 0.8 | 2.0 | 0.5 | 2.3 | 0.7 | 3.4 |
| ***Characteristics*** |  |  |  |  |  |  |
| *Flush toilet/pit latrine* | 63.9 | 8.8 | 79.9 | 20.8 | 81.1 | 36.2 |
| *High quality house material* | 66.0 | 19.0 | 81.2 | 28.8 | 84.5 | 41.3 |
| *LPG/Electricity for cooking* | 47.7 | 5.3 | 59.6 | 8.3 | 79.3 | 23.4 |
| *Piped/handpump water source* | 92.6 | 72.3 | 92.3 | 81.1 | 86.4 | 84.6 |

Sources: NFHS 2 report; NFHS 3 report; NFHS 4 report

Table A2. Percentage of the full women’s sample (including pregnant and never-married women) in each strata of the SEP exposures and the outcome

|  | ***Most developed states*** | | | ***Least developed states*** | | |
| --- | --- | --- | --- | --- | --- | --- |
|  | ***NFHS 2*** | ***NFHS 3*** | ***NFHS 4*** | ***NFHS 2*** | ***NFHS 3*** | ***NFHS 4*** |
| ***No Education*** | 31.73 | 18.13 | 15.35 | 62.71 | 40.17 | 34.81 |
| ***Primary*** | 18.68 | 13.22 | 11.10 | 14.30 | 12.92 | 12.72 |
| ***Secondary*** | 36.19 | 55.48 | 57.52 | 16.09 | 36.89 | 42.66 |
| ***Higher*** | 13.40 | 13.17 | 16.02 | 6.90 | 10.02 | 9.81 |
| ***Low SoL*** | 44.62 | 29.29 | 8.72 | 69.45 | 53.12 | 29.15 |
| ***Middle SoL*** | 39.60 | 35.66 | 26.36 | 24.81 | 28.23 | 38.76 |
| ***Higher SoL*** | 15.79 | 35.05 | 64.91 | 5.73 | 18.65 | 32.09 |
| ***Overweight*** | 17.76 | 19.26 | 22.61 | 5.89 | 11.88 | 14.86 |

Table A3. Predicted prevalence of overweight from the regression model in India’s least developed states (using the full sample of women including pregnant and never married women)

|  |  | ***Urban*** | | | ***Rural*** | | |
| --- | --- | --- | --- | --- | --- | --- | --- |
| ***Survey*** | ***Education*** | ***Prevalence*** | ***Lower*** | ***Upper*** | ***Prevalence*** | ***Lower*** | ***Upper*** |
| ***2005-06*** | ***No Education*** | 14.60 | 12.87 | 16.32 | 3.50 | 3.15 | 3.85 |
|  | ***Primary*** | 16.42 | 13.92 | 18.92 | 5.06 | 4.30 | 5.82 |
|  | ***Secondary*** | 18.37 | 16.67 | 20.06 | 6.38 | 5.70 | 7.06 |
|  | ***Higher*** | 27.61 | 24.71 | 30.51 | 11.62 | 8.87 | 14.36 |
| ***2015-16*** | ***No Education*** | 27.09 | 25.83 | 28.36 | 11.09 | 10.66 | 11.52 |
|  | ***Primary*** | 25.31 | 23.83 | 26.78 | 12.03 | 11.45 | 12.61 |
|  | ***Secondary*** | 23.70 | 22.72 | 24.68 | 10.97 | 10.55 | 11.40 |
|  | ***Higher*** | 28.80 | 27.60 | 30.00 | 14.45 | 13.67 | 15.24 |
